# Genetic engineering of hematopoiesis: current stage of clinical translation and future perspectives

**DOI:** 10.15252/emmm.201809958

**Published:** 2019-01-22

**Authors:** Luigi Naldini

**Affiliations:** ^1^ San Raffaele Telethon Institute for Gene Therapy IRCCS San Raffaele Hospital and Research Institute “Vita ‐ Salute San Raffaele” University Medical School Milan Italy

**Keywords:** gene editing, gene therapy, hematopoietic stem cells, lentiviral vectors, transplantation, Genetics, Gene Therapy & Genetic Disease, Haematology, Stem Cells

## Abstract

Here I review the scientific background, current stage of development and future perspectives that I foresee in the field of genetic manipulation of hematopoietic stem cells with a special emphasis on clinical applications.

## Introduction

**Figure 1:**
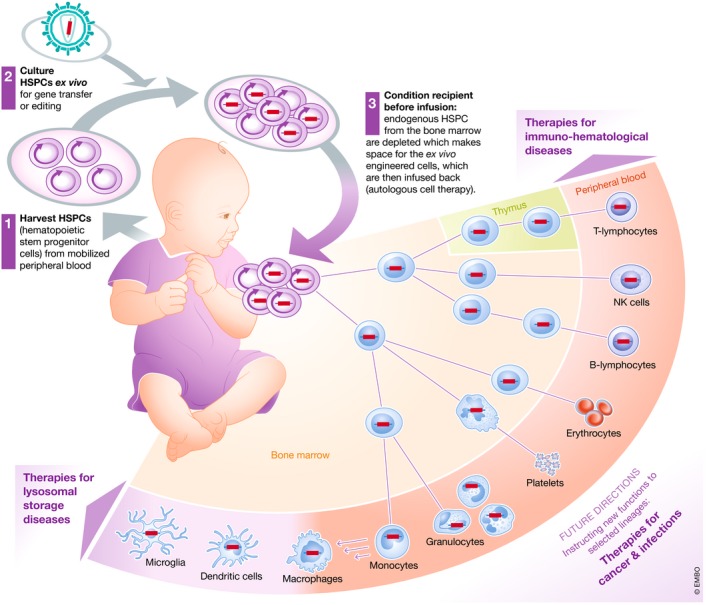
HSC gene therapy A schematic representation of HSC GT showing the crucial steps of the process and its potential clinical applications: (1) HSPC are harvested from the mobilized peripheral blood or bone marrow of a patient and (2) cultured *ex vivo* in suitable conditions allowing maintenance or expansion of the rare cells with long‐term repopulating potential, while they are subjected to gene transfer or gene editing. The patient is then administered a conditioning regimen which depletes endogenous HSPC from the bone marrow and makes space for her/is *ex vivo* engineered cells, which are then infused back (autologous cell therapy). The gene‐modified cells engraft in the bone marrow, where they self‐renew potentially for the lifetime of the individual while giving rise to differentiating progeny along all hematopoietic lineages. The mature gene corrected cells repopulate vascular and extravascular compartments with functional cells that can reverse pre‐existing pathologies affecting the lymphoid system, such as primary immunodeficiencies, the erythroid lineages, such as thalassemia and sickle cell disease, scavenger cells of myeloid lineage found throughout peripheral organs and, in part, the central nervous system and suffering from storage disease due to a lysosomal enzyme deficiency. As we are becoming confident with the safety and efficacy of genetic engineering of hematopoiesis, new applications are also explored which, rather than replacing inherited defective genes, are instructing new functions to selected lineages to better fight cancer or chronic infections.

Hematopoietic stem cell gene therapy (HSC GT) is becoming a powerful and versatile strategy to treat a growing number of human diseases. Hematopoietic stem/progenitor cells (HSPC) are harvested from the body, genetically engineered *ex vivo,* and re‐infused into the same individual after administration of a conditioning treatment that favor their engraftment in the bone marrow. The engrafted HSPC ensure a steady supply of genetically engineered progeny potentially for the recipient's lifetime. Mature cells of different lineages may then reverse pathological conditions such as inherited immune deficiencies, blood and storage disorders, infections, and cancer (Fig 1).

### From early HSC gene therapy testing to the development of lentiviral vectors

Whereas HSC GT has been one of the earliest and longest sought application of gene therapy, its clinical development followed a long and bumpy road, probably defining better than any other the course of the whole gene therapy field. HSC GT started with premature and overly hyped expectations, soon faced unexpected hurdles and toxicity derailing its course and only recently reached safe and successful clinical results in a growing number of diseases thanks to an improved generation of tools (Naldini, 2011, 2015; Dunbar *et al*, 2018).

Successful HSC GT requires inserting the therapeutic gene into the cellular chromosomal DNA, or editing a selected nucleotide sequence, to ensure maintenance of gene correction as the cell replicate its genome and transmit it to the progeny, whether during self‐renewal or in the output of differentiating cell lineages. The most commonly used gene transfer tools rely on the ability of retroviruses to integrate into the chromatin of infected cells and are obtained by engineering the virus into replication‐defective vehicles (vectors) of self‐contained gene expression cassettes (Kay *et al*, 2001; Naldini, 2011). Early HSC GT trials proved the remarkable therapeutic potential of this strategy in some primary immunodeficiencies (PID) where a low input of gene corrected progenitors was sufficient to reconstitute a functional T‐cell compartment (Fischer *et al*, 2015; Ferrua & Aiuti, 2017). Unfortunately, some of these trials also reported delayed emergence of leukemia in a fraction of treated patients, whose origin was traced to a vector insertion near an oncogene resulting in activation of its tumorigenic potential (Hacein‐Bey‐Abina *et al*, 2008; Howe *et al*, 2008; Stein *et al*, 2010; Braun *et al*, 2014). The low efficiency of HSC gene transfer and the risk of genotoxicity associated with early generation γ‐retroviral vectors used in these trials prompted further research toward developing improved gene transfer tools with enhanced efficiency and safety.

Toward these goals, we have contributed over the past 25 years to develop a new gene transfer system based on the human lentivirus HIV, which has become widely used in biomedical research (Naldini *et al*, 1996, 2016). These lentiviral vectors (LV) exploit the core machinery of HIV for efficient nuclear translocation and integration of their genetic payload into the chromatin of human cells and are packaged with the surface envelope of other viruses to allow targeting a wide range of cells. Vector development throughout the years stripped them of HIV components contributing to pathogenesis but dispensable for gene transfer (Dull *et al*, 1998; Zufferey *et al*, 1998) and validated their safety and efficacy in several experimental assays and disease models. It was shown that LV, as compared to γ‐retroviral vectors, not only provide for higher efficiency of gene transfer, but also for a significantly decreased risk of genotoxicity, thanks to an advanced vector design and the HIV integration pattern in the cell genome, which alleviates the risk of occurrence of oncogene activating insertions (Modlich *et al*, 2009; Montini *et al*, 2009; Wang *et al*, 2009; Zhou *et al*, 2010; Biffi *et al*, 2011).

### Lessons from current HSC GT trials

LV first entered clinical testing in T cells of HIV‐infected patients to instruct viral resistance (Levine *et al*, 2006; Tebas *et al*, 2013) and, few years later, in HSC (Cartier *et al*, 2009). The rationale for the first‐in‐human testing of LV‐based HSC GT was built on the premise that high gene transfer efficiency would allow broadening application to the treatment of diseases conventionally treated with allogeneic HSC transplantation (HSCT), with enrollment offered to patients lacking a suitably matched HSC donor. The demyelinating leukodystrophies Adrenoleukodystrophy (ALD) and Metachromatic Leukodystrophy (MLD), and the combined immune and platelet deficiency Wiskott‐Aldrich syndrome (WAS) were the first diseases tested (Cartier *et al*, 2009; Aiuti *et al*, 2013; Biffi *et al*, 2013; Hacein‐Bey Abina *et al*, 2015). Today > 200 patients have been treated worldwide by LV‐based HSC GT for several diseases, including ALD (Eichler *et al*, 2017), MLD (Sessa *et al*, 2016); WAS (Biasco *et al*, 2016); chronic granulomatous disorder, some PID (De Ravin *et al*, 2016b); and mucopolysaccharidoses, thalassemia, and sickle cell disease (Cavazzana‐Calvo *et al*, 2010; Ribeil *et al*, 2017; Thompson *et al*, 2018; Marktel *et al*, 2019); Fanconi anemia (Rio *et al*, 2018); and HIV infection (DiGiusto *et al*, 2010), with a follow‐up reaching up to 10 years for the earliest treated patients. According to available disclosures, most patients treated with LV‐based HSC GT show safe establishment and stable long‐term hematopoietic reconstitution with a polyclonal graft comprising a substantial proportion of gene‐modified cells throughout all lineages, conceivably maintained by sizable numbers of engrafted transduced *bona fide* HSC. Lack of expanding or dominant clones enriched for vector insertion at oncogenes supports the predicted low risk of genotoxicity of LV insertion. Substantial correction of disease is observed in most treated patients, provided that sufficiently high levels of chimerism with gene‐modified cells and of transgene expression within the transduced cell progeny are concurrently achieved in the graft. If these positive clinical results continue to be reproduced in more patients and at longer follow‐up, several HSC GT currently in advanced clinical testing are expected to become registered drugs and eventually provide the standard‐of‐care for some genetic diseases. Because of the increasing confidence with its safety and therapeutic potential, clinical testing of LV‐based HSC GT has now moved from confined testing in otherwise lethal conditions to broader application wherever HSCT is considered as treatment option, and patient inclusion criteria are expanding from the lack of a suitably matched HSC donor to the rationale of potentially providing better efficacy and/or improved safety over allogeneic transplantation. Emerging trials of HSC GT are testing its potential for delivering biotherapeutics in some cancer conditions (Escobar *et al*, 2014, 2018), thus further expanding its application from the replacement of faulty inherited genes to cell and gene‐based delivery of transgene products and the instruction of novel functions to the engineered HSC and/or their progeny.

### Looking forward: prospective development of HSC gene therapy

HSC GT may eventually surpass allogeneic HSCT because of lower treatment related short‐ and long‐term morbidity and, in some cases, improved efficacy (Touzot *et al*, 2015; Sessa *et al*, 2016; Thrasher & Williams, 2017). Because HSC GT exploits autologous cells, it is virtually available to every patient, abrogates the risk of graft versus host disease, and does not need to overcome immunological barriers in the recipient (except, in some cases, to the therapeutic gene product), thus sparing the need for immune suppression in the recipient.

Autologous setting also allows exploring a range of reduced intensity conditioning. These maneuvers allow reducing the morbidity of the procedure as compared to standard allogeneic HSCT because of faster immune reconstitution, albeit this benefit is often counteracted by the impact of *ex vivo* culture and transduction on HSPC engraftment (see below). We are just beginning to capture all HSC GT advantages in the clinic, also considering that most conditioning strategies have been shaped in the context of immune mismatch between donor and recipient and may need adjustment for best proficiency in the autologous setting. New depleting drugs could be tested, which better target hematopoietic progenitors while sparing the stromal components of bone marrow. These strategies may allow rapidly establishing sufficient chimerism of modified progenitors without exposing the patient to the risk of neutrophil and/or lymphoid cytopenia/aplasia. If successful, these advances may alleviate the time and need for hospitalization and eventually transform HSC GT into an out‐patient treatment.

Furthermore, whether all cell populations in the administered product need to be gene modified or only a subset thereof, comprising the long‐term engrafting progenitors, may be explored and adjusted to the need of the condition (Baldwin *et al*, 2015; Zonari *et al*, 2017). One could envisage administering unmodified short‐term progenitors which, having been spared *ex vivo* culture and transduction, should ensure faster reconstitution, admixed with transduced HSC, which would progressively replace the graft with gene‐modified progeny (Zonari *et al*, 2017). On the other hand, were only short‐term progenitors transduced and administered, one might obtain a time‐limited graft of transduced cell possibly restricted to selected lineages, best fitting genetic engineering applications fighting cancer and infection. Whereas HSC GT has been performed until now with cells purified according to surface expression of the CD34 marker, we can envisage exploring new protocols that further enrich for the repopulating HSPC fraction by adding other markers to the selection profile, provided that clinically compliant technologies for multi‐parametric cell sorting coupled with advanced microfluidics become available (Notta *et al*, 2011; Fares *et al*, 2017; Radtke *et al*, 2017). HSC‐enriched cell products would allow saving on culture volume and vector need, enabling more cost‐effective industrialization of automated cell processing.

The potential advantages of HSC GT should be assessed in view of the continuous improvements in overall and disease‐free survival after allogeneic HSCT, which have been made possible by more refined donor typing and matching, purging of selected immune populations and even *ex vivo* HSPC expansion prior to transplantation, together with better prophylaxis and treatment of post‐transplant infections, using drugs, adoptive immunotherapy, and pathogen‐specific T‐cell clones (Li Pira *et al*, 2017). Allogeneic HSCT and HSC GT may mutually benefit from these progresses, which may eventually transform life‐saving but until now clinically demanding strategies into low‐morbidity and more broadly applicable procedures. As we improve the overall quality of the genetically engineered cell product, we may eventually phase‐out mutagenic conditioning drugs while phasing‐in biologicals that selectively act on resident HSPC and/or their niche to promote engraftment of the administered cells. This endeavor, long‐sought in the HSCT field, will be facilitated by HSC GT because partial donor cell chimerism may be more easily established in the autologous setting and be sufficient to achieve full therapeutic benefits in several diseases. Niche antagonists, HSPC‐depleting antibodies, and immunotoxins may all work better in the autologous setting and could find a favorable testing ground in HSC GT (Chhabra *et al*, 2016; Palchaudhuri *et al*, 2016).

One of the most intriguing possibility offered by genetic engineering versus donor HSC transplantation is increasing the therapeutic gene dosage above the wild‐type level, which is proving crucial in treating disease manifestations in the central and peripheral nervous system and, possibly, other organs of some lysosomal storage disorders, such as MLD (Biffi *et al*, 2006, 2013; Gentner *et al*, 2010; Visigalli *et al*, 2010). Here, HSC GT becomes a platform for both cell replacement and sustained tissue release of biotherapeutics. Microglia progenitors, which have been partly depleted by the chemotherapeutic conditioning (Capotondo *et al*, 2012), are replaced by the infused gene corrected HSPC and give rise to a steady supply of functional progeny, which not only clear the storage and prevent inflammatory activation but also release in part the functional enzyme for its uptake and metabolic cross‐correction of neighboring CNS‐resident cells. The more enzyme they produce from the integrated vector cassette, the more efficacious is the strategy, surpassing the limited benefits reported for HSCT from healthy donors. An unexpected advantage of cell‐based local delivery appears to be substantial efficacy despite a relatively minor extent of overall cell replacement achieved in the affected tissue. This benefit may reflect improved bioavailability because the biotherapeutics is locally released within the extracellular milieu of the tissue, bypassing the need for systemic biodistribution and extravascular access, which requires substantially higher input doses. The potential scope of this cell‐ and gene‐based delivery strategy may be further expanded to acquired diseases such as neurodegenerative diseases, cancers, and chronic infections, where selected hematopoietic populations may become smart vehicles of a biotherapeutics homing to the organs and tissue sites affected by the disease. One could envisage its application to the delivery of enzymes in the extracellular milieu to promote extracellular matrix remodeling, clearance of toxic deposits, but also of signals or cytokines promoting cellular survival and growth, or modulating immune response (Ben Nasr *et al*, 2017), either positively or negatively, or of factors mediating resistance to specific pathogens. In several such applications, HSPC‐mediated delivery may be designed to target the diseases sites by advanced engineering of the vector cassette, which could be selectively expressed in the lineage/cell state of choice, such as microglia, tumor‐infiltrating macrophages (Escobar *et al*, 2014), or cells responding to chemotactic cues toward specific pathogen‐infected areas. Local release of a biotherapeutics, rather than relying on its exogenous administration, spares off‐target tissues from exposure while reaching sustained expression at physiological levels in the target sites, thus limiting the risk of off‐target adverse events and, possibly target desensitization from exposure to excess or fluctuating doses.

### Outstanding challenges and further goals ahead

Whereas clinical deployment of LV‐based HSC GT has been consistently safe, the extent of gene transfer into repopulating HSC and the proportion and time course of establishment of the transduced cells graft have sometimes been limiting and highly variable among trials and even patients treated within the same protocol, highlighting the impact of variables that currently escape our understanding or control. For instance, in ongoing HSC GT trials of β‐thalassemia and sickle cell disease, limited transduction rate has been identified as a major hurdle to achieve consistent and robust therapeutic benefit, i.e., transfusion independence, in most treated patients (Thompson *et al*, 2018; Marktel *et al*, 2019).

Among the factors that affect HSC gene transfer are the vector type and design, the quality and infectivity of the vector batch, the transduction protocol and the age and healthy versus diseased condition of the HSPC donor. As compared to other cell types, HSC are poorly permissive to LV gene transfer, due to quiescence and the presence or enhanced expression of innate or adaptive restriction factors acting at different steps of the transduction pathway (Santoni de Sio *et al*, 2006; Wang *et al*, 2014). Robust gene transfer thus requires cell pre‐stimulation with activating cytokines and high concentrations of LV with high specific infectivity. Vector entry may occur from the plasma membrane or from endosomes following phagocytosis, as dictated by the choice of envelope pseudotype, and is subject to differential restriction. Upon entering the cytosol, the vector core undergoes partial uncoating while engaging in reverse transcription and translocation to the nucleus. These steps are sensitive to a combination of cellular effectors and antagonists, resulting in a variable but generally small fraction of the up‐taken cores reaching the nucleus. Depending on the target cell type, some of the best‐known factors affecting these steps include the uncoating co‐factors cyclophilin A and CPSF6, cytosolic sensors of exposed nucleic acids, such as cGAS and AIM2, and enzymes controlling the availability of a balanced deoxynucleotide pool to support reverse transcription, such as SAMHD1 (Kajaste‐Rudnitski & Naldini, 2015). More recently, IFN‐inducible antiviral factors, such as IFITM3, have been shown to impair LV entry into HSPC according to donor‐dependent expression level (Petrillo *et al*, 2018). As more restriction factors are uncovered, new drugs become available to counteract them and enable enhanced (Wang *et al*, 2014; Petrillo *et al*, 2015; Zonari *et al*, 2017; Heffner *et al*, 2018) and better matched gene transfer among donors, as in the case of cyclosporin H, which overcomes IFITM3 restriction (Petrillo *et al*, 2018).

Another set of variables that affect the transduced cells graft are the administered dose of repopulating cells and the extent of cellular depletion achieved in the bone marrow by the conditioning regimen, whose pharmacokinetic and pharmacodynamic may vary among individual patients. The HSPC source, with mobilized peripheral blood now preferred over bone marrow, and the mobilization protocol affect the total amount and proportion of long‐term repopulating versus committed progenitor cells in the harvest. Current efforts toward increasing the yield of transduced cells comprise more effective mobilization and collection regimes, using antagonists of the CXCL12/CXCR4 axis, such as Plerixafor (Broxmeyer *et al*, 2005), and of the VLA‐4/VCAM‐1 axis (Ghobadi *et al*, 2018), which mediate HSPC homing and retention in the marrow niche, and/or granulocyte colony‐stimulating factor (G‐CSF) and CXCR2 agonists, such as GROβ, acting on neutrophils and resulting in MMP‐9 secretion, matrix digestion and HSPC release (Hoggatt *et al*, 2018), and *ex vivo* expansion of the collected HSPC (see below).

Many diseases impact on the HSPC and their bone marrow niche. For instance, HSC may accumulate DNA damage or replication stress because of excess demand posed by chronic infection, lack or dysfunction of individual lineages, enhancing susceptibility to *ex vivo* culture and gene transfer, as well as decreasing engraftment capacity (Flach *et al*, 2014). The same and other conditions may also modify the composition of the bone marrow population, the relative proportion, and lineage commitment of different progenitors. The non‐hematopoietic components of the bone marrow niche may also be affected by disease, directly or through cross‐talk with the altered hematopoietic populations and provide a less favorable environment for engraftment and reconstitution by the infused gene‐modified HSPC. The deployment of HSC GT should thus be suitably adjusted when the bone marrow health is compromised by disease, for instance by pre‐treating the underlying condition (i.e., alleviating storage in lysosomal storage disorders by enzyme replacement therapy, iron overload, and erythroid lineage expansion in hemoglobinopathies, inflammation in immunodeficiencies).

We still lack robust predictors of the treatment outcome when administering HSC GT, as the HSPC content and vector copy number *per* cell genome (VCN; a measure of the extent of transduction) in the cell product often but not always correlate positively with the extent and duration of the transduced cell graft. This is likely because only a minute fraction of HSC among the cultured cells contributes to the graft, with different cell subsets contributing to early versus late phase of the graft. These rare cell subsets may respond differently to the culture conditions and gene transfer, and their response may be difficult to readout because of the confounding effect of the excess downstream progenitors and differentiating cells growing in the culture. As we better characterize surface markers of HSPC in culture, we could envisage assessing VCN in a prospectively identified subset of engrafting HSC in the product, provided that the employed markers are not altered by the *ex vivo* culture.

As mentioned above, *ex vivo* culture and stimulation of HSPC, while being needed for efficient gene transfer, may adversely impact their engraftment and long‐term repopulation capacity (Mazurier *et al*, 2004; Piras *et al*, 2017; Zonari *et al*, 2017). Indeed, HSC GT trials often report delayed engraftment of myeloid cells and platelets as compared to conventional HSCT, a serious drawback which is addressed by increasing the dose of administered cells as much as feasible, and investigating novel administration routes such as intra‐osseous, which bypasses the splenic filter (Mazurier *et al*, 2003; Marktel *et al*, 2019). HSPC culture conditions have been continuously improved as we moved away from serum‐containing to fully defined media, and novel compounds and signaling pathways are discovered that better maintain or expand HSC in culture (Boitano *et al*, 2010; Cutler *et al*, 2013; Watts *et al*, 2013; Horwitz *et al*, 2014; Wagner *et al*, 2016; Fares *et al*, 2017; Zonari *et al*, 2017). We still lack, however, a robust protocol for expanding long‐term repopulating HSC from bone marrow or mobilized peripheral blood *ex vivo*, thus currently most strategies aim to limit culture time and extent of stimulation to the minimal requirements for achieving the desired level of transduction and exploiting, where necessary, transduction enhancing compounds. It is also likely that preserving short‐term but rapidly engrafting progenitors and long‐term repopulating HSC pose different requirements and can thus be hardly optimized in the same culture. Cell separation, admixing of differently processed products, including expanded cell fractions, may eventually be the way to attain the most rapid as well as durable hematopoietic reconstitution in HSC GT. Meanwhile, research will continue to explore new and emerging strategies to better reproduce *ex vivo* the crucial biological components of the bone marrow niche supporting HSC self‐renewal, i.e., by co‐culturing HSPC with mesenchymal stromal cells (de Lima *et al*, 2012) and endothelial cells (Hadland *et al*, 2015), or to generate bona fide HSC from pluripotent stem cells (iPSC/ESC) (Gori *et al*, 2015; Sugimura *et al*, 2017) or endothelial cells (Lis *et al*, 2017) to provide an unconstrained HSC source, particularly important when underlying disease or conditions have exhausted the HSC of the patient.

Genetic modification, whether by vector‐mediated gene transfer or nuclease‐mediated gene editing (see below), may trigger intracellular responses in HSPC affecting proliferation, differentiation, and overall fitness, whether transiently or long‐term (Rossetti *et al*, 2011; Piras *et al*, 2017). Such responses are dependent on sensors, in the cytoplasm and nucleus, of exogenous nucleic acids as well of DNA breaks, fragments, and free ends, which may trigger a DNA damage response (DDR) (Ciccia & Elledge, 2010), with activation of p53‐dependent cell cycle arrest, senescence or apoptosis, as well as an interferon response, with ensuing HSPC activation and myeloid commitment, which may exhaust long‐term repopulation potential (Beerman *et al*, 2014). Perfecting genetic engineering technologies to prevent or counteract nucleic acid sensing and the downstream responses may be important to ensure long‐term hematopoietic reconstitution of the recipient by genetically engineered HSC.

A major concern remains the long‐term genotoxicity of quasi‐random genome‐wide integration of LV. Whereas this risk has not surfaced in the clinic to this date as a pathological finding, we must remain aware of its possible occurrence and continue our efforts toward further alleviating its risk. Several conditions may decrease the risk that a gain‐ or loss‐of‐function in a growth controlling gene in a transduced cell progresses to transformation, including the status of that cell in the hematopoietic hierarchy, the proliferation demand posed on that cell *in vivo*, the competition for engraftment, growth and repopulation with other transduced cells, the presence of selective pressure that may enhance its growth advantage, or of tumor promoting conditions, such as chronic inflammation (Cooper *et al*, 2017). The current design of LV alleviates but does not fully abrogate the risk that a rare insertion may transcriptionally activate and/or truncate an oncogene or disrupt a tumor‐suppressor gene by promoter insertion or aberrant splicing (Biffi *et al*, 2011; Cesana *et al*, 2014). Improved vector designs addressing one or more of these adverse outcomes have been proposed and one wonders whether their clinical testing should await actual emergence of the risk they are meant to address or start on the bases of pre‐clinical studies in models that amplify the risk of genotoxicity.

All limitations and adverse responses to HSPC gene transfer discussed above may contribute to determine oligoclonal composition and premature clonal exhaustion in the transduced cell graft. These conditions are risk factors for the emergence, selection, and expansion of abnormal clones bearing gain‐of‐function mutations acquired because of vector insertion or spontaneously during *ex vivo* cell culture or from residual recipient cells exposed *in vivo* to the mutagenic effect of chemotherapy. Longitudinal clonal tracking of the reconstituted graft in HSC GT therapy trials thus represents an important line of investigation to firmly establish the safety and efficacy of the strategy. Because LV insertion occurs semi‐randomly, each transduced cell acquires a unique genetic marker represented by the joined vector sequence to the flanking genomic sequence, which may be used to track its progeny *in vivo* (Gabriel *et al*, 2009; Berry *et al*, 2014). By vector insertion analysis, the behavior and lineage output of individual gene‐modified HSC can be longitudinally tracked in GT patients. These studies are providing an unprecedented granular insight into the population dynamics of hematopoiesis post‐transplant and are challenging predictions based on murine models and xenotransplantation studies. Recent studies report that *ex vivo* activated and proliferated HSC may return to short‐ or long‐term quiescence *in vivo*, while more committed progenitors, or a fraction of the infused HSC, provide for early and rapid reconstitution of the recipient hematopoiesis in an emergency‐driven modality, before steady‐state hematopoiesis driven by long‐term self‐renewing HSC eventually establishes (Biasco *et al*, 2016; Scala *et al*, 2018). As we are revising our understanding of the hierarchical relationship among hematopoietic progenitors engaged in lineage commitment and a picture emerges of a quasi‐continuum of states able to transition from undifferentiated stem cell to mono‐lineage commitment (Notta *et al*, 2016; Velten *et al*, 2017; Belluschi *et al*, 2018), we must question whether the different repopulation modalities observed early versus late post‐transplant are facultative options of a plastic HSC, or represent the activity of different types of HSPC intrinsically wired for short‐ versus long‐term reconstitution. It would also be important to assess whether *ex vivo* culture primes some HSPC for emergency repopulation drive or differentially affect defined subsets of HSPC. Insights into this fascinating biology may guide better cell manipulation and improve our prediction on the *in vivo* behavior of administered cells.

Another route to better vector design concerns improving the stringency of transgene expression control to better reproduce the endogenous pattern of expression of the replaced gene and sharply target the desired cell lineage. These aims are undertaken exploiting transcriptional control sequences derived from the endogenous affected gene, and by the inclusion of microRNA target sequences in the transgene transcript which prevent its ectopic or aberrant expression in off‐target cell types expressing the cognate microRNA (Brown *et al*, 2007; Gentner *et al*, 2010).

Finally, even within the setting of autologous HSC GT we should be aware of the potential emergence of immune response to the therapeutic gene product, which may act as foreign antigen in the recipient, and to the transduced cells, which may transiently express after transduction vector‐derived components, such as allogeneic HLA molecules incorporated into the virions when budding from the plasma membrane of the human LV producer cell (Milani *et al*, 2017). Although the occurrence of such immune complications has not been reported yet in clinical trials, we should closely watch for them as the application of HSC GT broadens to new diseases and exploits milder conditioning regimens where an immune response may be more easily encountered. Were such responses be observed and have detrimental impact on HSC engraftment, one could exploit a range of immune suppression strategies, whether broadly acting or transgene‐specific, to overcome them and ensure graft take and maintenance.

### Precision genetic engineering: targeted gene editing

Whereas LV have provided the means to improve the risk/benefit ratio and broaden the applications of HSC GT, it remains challenging to reconstitute physiological expression in every transduced cells when replacing genes by random integration of an expression cassette of constrained size. Aberrant expression may pose a safety risk when the replaced gene controls cell growth. Moreover, a residual genotoxic risk remains associated with genome‐wide vector insertion, despite the advances in its design. More precise strategies of genetic engineering based on targeted genome editing have the potential to overcome these hurdles by allowing *in situ correction of mutant genes* and targeted integration of transgene cassettes into *safe genomic harbors* (Urnov, 2018). *In situ* correction may stringently reconstitute both function and physiological expression control of mutant defective genes. Targeted integration into a safe genomic harbor, empirically identified as tolerant to insertion and permissive to robust and consistent transgene expression without adverse effects on the nearby or distant genome, allows reconstitution of gene expression at predictable and homogeneous levels in nearly all transduced cells (Lombardo *et al*, 2011). Both approaches limit the genotoxic risk to the small fraction of genome which may be targeted, whether intentionally or because of off‐target activity, by the chosen designer endonuclease.

Targeted genome editing is based on delivering a double‐stranded DNA break (DSB) by a designer endonuclease to a chosen and unique sequence of the genome and exploiting distinct repair pathways of that DSB for the intended editing outcome. If the DSB affects a coding or essential regulatory sequence and the repair occurs by non‐homologous end joining (NHEJ), an error prone pathway available to most cell types and throughout all cell cycle phases, the outcome may be disruption of the encoded function. In HSC GT, this may be exploited for instance to silence a repressor of the fetal globin gene in the erythroid lineage and rescue by its compensatory expression the hemoglobin defect in β‐thalassemia or sickle cell disease (Orkin & Bauer, 2019). If instead the DSB is repaired by the homology‐dependent recombination (HDR) pathway, which is restricted to cells engaged in S/G2 phases of the cell cycle, and an exogenous repair DNA template is provided carrying the intended sequence framed by homology to the DNA flanking the break site, the outcome may be conversion of the targeted sequence to the provided one (editing). The latter strategy may be used for correcting individual mutations or, more often, for targeted insertion of a functional cDNA version of the mutant gene downstream its endogenous promoter (Lombardo *et al*, 2007), or of a whole transgene expression cassette into a safe genomic harbor (Lombardo *et al*, 2011).

Designer DNA endonucleases targeting the intended sequence can be generated using several platforms, which consist of sequence‐specific DNA recognition modules combined with endonuclease domains. Zinc‐finger, TALEN, and meganuclease platforms exploit dimeric or monomeric protein‐based DNA sequence recognition to tether an endonuclease on the target DNA, while the more recently developed CRISPR/Cas systems exploit RNA‐based sequence recognition coupled to activation of the protein endonucleolytic activity (Doudna & Charpentier, 2014). Current data support the contention that all platforms may be optimized to achieve robust and highly specific cleavage of the intended target DNA with limited off‐target activity. Given the wide choice of platforms and the continuously emerging variants with novel or expanded target sequence recognition properties, improved activity and specificity, one may assume that virtually every sequence uniquely occurring in the genome might be efficiently and specifically targeted (Tsai & Joung, 2016; Urnov, 2018).

Because HSPC engineering is currently performed *ex vivo*, delivery of the machinery required for targeted genome editing can be satisfactorily achieved. Today, this is most often done by electroporating purified nuclease ribonucleoprotein complexes (i.e., Cas nucleases preassembled with single guide RNA) or nuclease‐encoding mRNAs (for the other platforms), followed, when required, by the delivery of the repair template through a viral vector, such as adeno‐associated virus‐derived (AAV6) or integrase‐defective LV (IDLV) (Genovese *et al*, 2014; Wang *et al*, 2015; Dever *et al*, 2016; De Ravin *et al*, 2016a, 2017; Schiroli *et al*, 2017). Whereas AAV6 and IDLV can shuttle relatively large DNA molecules into the nucleus, thus enabling large edits and insertions, co‐electroporation of short single‐ or double‐stranded oligonucleotides with the nuclease can instead be used for editing few base pairs. All these approaches have the advantage to achieve a high but transient spike of nuclease expression in the cultured cells, thus limiting its activity to a small window of time and, consequently, alleviating toxicity and off‐target activity. The major hurdle to exploit targeted gene editing in HSC is that procedures based on HDR suffer from limited efficiency in the most primitive progenitors, likely due to low expression and proficiency of the HDR machinery, cell quiescence, and limited uptake of repair template (Genovese *et al*, 2014). We and others could overcome at least in part these barriers by extending culture in conditions that drive even the most primitive cells into replication while preserving their engraftment capacity and by tailoring the gene editing machinery to avoid triggering innate immune cellular responses (Genovese *et al*, 2014; Wang *et al*, 2015; De Ravin *et al*, 2016a, 2017; Dever *et al*, 2016; Schiroli *et al*, 2017). Yet, the extent of HDR‐mediated DSB repair still decreases as we move from the most committed to the most primitive HSPC in culture, resulting in hematopoietic grafts of xeno‐transplant recipients comprising ≤ 20% HDR‐edited cells in the long term and showing oligoclonal composition. Whereas these levels of editing may be sufficient to treat some PID, such as SCID‐X1, in which a relatively small fraction of functional progenitors in the administered cell product nearly completely rescued the T‐cell deficiency in mouse models and in early gene therapy trials performed with relatively inefficient γ‐retroviral vectors (Fischer *et al*, 2015; Schiroli *et al*, 2017), they may be limiting in other applications, where more robust correction is needed, particularly of long‐term repopulating HSC.

Because nuclease‐mediated gene editing strictly depends on inducing DNA DSB, the concern was raised whether such breaks, albeit limited to few or even a single genomic site, trigger a DNA damage response (DDR) in the edited HSPC with the potential of inducing adverse effects on their key functional properties, such as long‐term hematopoietic repopulation (Ciccia & Elledge, 2010; Milyavsky *et al*, 2010; Biechonski *et al*, 2018; Conti & Di Micco, 2018). We recently investigated the transcriptional and functional impact of gene editing on HSPC and found that even a single DSB triggers a detectable transcriptional DDR with p53 pathway activation, p21 induction, and delayed cell proliferation (Schiroli *et al*, 2019). The response was independent on the platform used for DSB induction and similar at different targeted loci. On the other hand, the extent and time course of DDR were strictly dependent on nuclease specificity; i.e., the total DSB load delivered to the treated cells. Nucleases of lesser specificity, which cleaved DNA at the target and few other off‐target sites, resulted in robust and prolonged DDR induction, halted proliferation up to irreversible arrest and loss of clonogenic activity. Highly specific nucleases, on the other hand, with cleavage virtually detectable only at the target site, triggered a transient and fully reversible DDR, whose only impact appeared to be a lower yield of edited versus untreated repopulating cells, because of the transient proliferation delay imposed on the former by the DSB repair. These findings suggest that, provided that highly optimized reagents and protocols are used, seamless targeted gene editing should be feasible in human HSPC and conceivably compatible with preservation of their long‐term repopulation capacity, although only clinical testing in humans, now well on its way, will ultimately prove it.

Ongoing research on HSC gene editing aims to further improve the efficiency of HDR in the most primitive cells, i.e., by favoring HDR versus NHEJ or customizing template design and enhancing its tethering to the repair site (Richardson *et al*, 2016), and to increase the yield of edited cells, i.e., by transiently inhibiting the p53 response (Schiroli *et al*, 2019). *In vitro* selection of edited HSC might also compensate for a low editing efficiency as it would allow obtaining a virtually pure population of edited cells that can be administered or further expanded before transplantation. Other approaches being investigated dispense of the need of inducing DNA DSB, such as editing mutations by base editors (Rees & Liu, 2018) and silencing genes or regulatory sequences by hit‐and‐run delivery of designer epigenetic modifiers, which instruct an inheritable repressive chromatin configuration on the targeted locus (Amabile *et al*, 2016). As these studies progress and we learn from the follow‐up of the first clinical testing of gene editing, we expect that an increasing number of genetic engineering options will become available when contemplating HSC GT and that each treatment could be optimized to the specific features of the underlying disease and, possibly, affected individual.

### Reaching the market

None of the promises of HSC GT spelled out above could be realized or become accessible to patients worldwide without the involvement of pharmaceutical industry and the processes of drug development, registration, and commercialization. Despite relying on an early γ‐retroviral vector design, HSC GT for one type of PID, Adenosine Deaminase Severe Combined Immunodeficiency (ADA‐SCID), has uniquely and consistently proven safe and has become the first *ex vivo* GT drug now available on the EU market (Aiuti *et al*, 2017). This milestone achievement proves that the challenges posed by commercial deployment of personalized therapies entailing complex *ex vivo* processing of autologous cells can be successfully met even in the setting of rare diseases when there is evidence of strong benefits to the patients. Several of the LV‐based HSC GT mentioned above are expected to reach the market in one or few years ahead. Our early experience in the field suggests that the most favorable path to further clinical development and market access of HSC GT is an early alliance between academic institutions that pioneered clinical testing and pharmaceutical companies.

The challenges faced by these types of GT‐based advance therapy medicinal products (ATMPs) when reaching the market are cost sustainability and fair public access. One acknowledges that the market cost of these therapies might be very high, considering: (i) the likelihood of providing robust therapeutic benefits up to a “cure”, resulting in dramatic improvement in survival and quality of life; (ii) the once‐in‐a‐lifetime nature of the treatment, which entails substantial saving on conventional symptomatic treatments otherwise constantly required by the untreated condition; (iii) the complexity of product manufacturing and its highly personalized nature, requiring individual processing of each patient's own cells; (iv) the need to reward industry investments in an area where preferential applications to rare diseases and the novelty and complexity of the drug challenge financial returns, especially in the short term. Whereas all these factors might justify the high costs of the first *ex vivo* GT, as their commercial deployment continues one expects that scale‐up and industrialization of product manufacturing and distribution will allow cost saving, also thanks to common exploitation across multiple applications. Moreover, once these therapies have become established and expand to new applications, we might envision designing together with regulatory authorities a more rapid and cost‐effective track to registration, which takes advantage of the previous clinical validation of the genetic engineering platforms used, whether based on gene transfer or editing, and limit the burden of pre‐clinical testing to address the novel and specific features of the new drug product. Because these ATMPs comprise multiple components (in the case of HSC GT: the gene transfer vector batch, an heterogeneous patient‐specific cell harvest, the transduction process), we should also avoid constraining by standard regulatory requirements the incorporation of incremental improvements to either step/component of a registered product, also considering that most improvements in platform efficiency and safety are likely to work across different applications and could be satisfactorily cross‐validated.

In conclusion, we are at the dawn of a new era in medicine, when many treatments such as the HSC GT discussed here challenge the traditional framework of drug development and require creative solutions to grant sustainable commercialization and fair public access, but also offer the reward of dramatic therapeutic benefits. Only a newly forged cooperation among all stakeholders in science, medicine, pharma industry, advocacy groups, regulatory and decision‐making bodies across nations, grounded on scientific evidence and inspired by the ethical principles of a just society will enable addressing these challenges and capturing the promise of providing long‐awaited relief to some of the unmet disease burden on our human brotherhood.

## Conflict of interest

LN is an inventor on pending and issued patents on LV technology, miRNA‐regulated LV, and targeted genome editing filed by the Salk Institute, Cell Genesys, Telethon Foundation, and/or San Raffaele Scientific Institute. According to the respective institutional policies, inventors may be entitled to receive some financial benefits from the licensing of such patents. In 2010, SR‐Tiget entered into a strategic alliance with GlaxoSmithKline (GSK) for the development up to marketing authorization of HSC GT for some rare diseases, starting with ADA‐SCID GT. Whereas SR‐Tiget remained responsible for pre‐clinical development and early clinical testing of all other therapies, GSK had option rights once clinical proof‐of‐concept was achieved. In 2014, MLD and WAS GT were licensed to GSK and GSK became the financial sponsor of the trials. In 2017, HSC GT for beta‐thalassemia was similarly licensed to GSK. Telethon and San Raffaele Scientific Institute are entitled to receive milestone payments and royalties upon commercialization of such therapies. In 2018, GSK transferred its portfolio to Orchard Therapeutics, which acquired the same rights and responsibilities of GSK on the further development of the program. SR‐Tiget had research collaboration on targeted genome editing in hematopoietic cells for treating some rare diseases with Sangamo Therapeutics and Editas Medicine. LN is a founder, owns equity, chairs the scientific advisory board, and is a consultant of Genenta Science, a biotechnology startup aiming at developing alpha‐IFN gene therapy of some types of tumors by tumor‐infiltrating monocytes. LN is a founder, owns equity and is a member of the scientific advisory board of Magenta Therapeutics, and is a member of the scientific advisory board of Oncorus and Sangamo Therapeutics.
